# Elasmobranch microbiomes: emerging patterns and implications for host health and ecology

**DOI:** 10.1186/s42523-021-00121-4

**Published:** 2021-09-15

**Authors:** Cameron T. Perry, Zoe A. Pratte, Ana Clavere-Graciette, Kim B. Ritchie, Robert E. Hueter, Alisa L. Newton, G. Christopher Fischer, Elizabeth A. Dinsdale, Michael P. Doane, Krystan A. Wilkinson, Kim Bassos-Hull, Kady Lyons, Alistair D. M. Dove, Lisa A. Hoopes, Frank J. Stewart

**Affiliations:** 1 School of Biological Sciences, Georgia Institute of Technology, Atlanta, GA USA; 2 Department of Microbiology and Immunology, Montana State University, Bozeman, MT USA; 3 Department of Natural Sciences, University of South Carolina Beaufort, Beaufort, SC USA; 4 Sharks and Rays Conservation Research Program, Mote Marine Laboratory, Sarasota, FL USA; 5 OCEARCH, Park City, UT USA; 6 Disney’s Animals, Science and Environment, Orlando, FL USA; 7 Marine Science Research Institute, Jacksonville University, Jacksonville, FL USA; 8 College of Science and Engineering, Flinders University, Bedford Park, SA Australia; 9 Chicago Zoological Society’s Sarasota Dolphin Research Program ℅ Mote Marine Laboratory, Sarasota, FL USA; 10 Research and Conservation Department, Georgia Aquarium, Atlanta, GA USA

**Keywords:** Bacteria, Sharks, Rays, Skates, Fishes, Health

## Abstract

Elasmobranchs (sharks, skates and rays) are of broad ecological, economic, and societal value. These globally important fishes are experiencing sharp population declines as a result of human activity in the oceans. Research to understand elasmobranch ecology and conservation is critical and has now begun to explore the role of body-associated microbiomes in shaping elasmobranch health. Here, we review the burgeoning efforts to understand elasmobranch microbiomes, highlighting microbiome variation among gastrointestinal, oral, skin, and blood-associated niches. We identify major bacterial lineages in the microbiome, challenges to the field, key unanswered questions, and avenues for future work. We argue for prioritizing research to determine how microbiomes interact mechanistically with the unique physiology of elasmobranchs, potentially identifying roles in host immunity, disease, nutrition, and waste processing. Understanding elasmobranch–microbiome interactions is critical for predicting how sharks and rays respond to a changing ocean and for managing healthy populations in managed care.

**Supplementary Information:**

The online version contains supplementary material available at 10.1186/s42523-021-00121-4.

## Introduction

Animal microbiomes influence host physiology, behavior, and evolution, yet have been studied sparingly in most fishes, including elasmobranchs (sharks, skates and rays). Understanding elasmobranch microbiomes is emerging as a research priority given the biological and ecological significance of this major vertebrate lineage. Representing over 1130 species, elasmobranchs occur in marine and freshwater habitats across the globe [1]. As carnivores, elasmobranchs shape food webs and move large amounts of carbon and energy through diverse feeding modes. While most elasmobranchs are generalist predators and feed intermittently, others such as the whale shark (*Rhincodon typus*) or basking shark (*Cetorhinus maximus*) are filter feeders, with diets more akin to those of baleen whales (suborder *Mysticeti*). Despite their diversity and ecological significance, nearly 50% of elasmobranch species are listed as “data deficient” by the International Union for the Conservation of Nature (IUCN) Red List, meaning that information is missing to fully assess their status [2]. For these taxa, we lack basic information on life history, physiology, and inter-species interactions, including those with microorganisms.

Elasmobranchs have traits that suggest unique interactions with microbes. Diverse bacteria are regularly cultured from the blood of healthy individuals [3], raising the question of why these microbes do not trigger an immune response. Indeed, while natural mortality events are rarely investigated and diagnosing elasmobranch disease remains challenging, elasmobranchs appear to be relatively disease-free [4]. Documented cases of cancer in elasmobranchs are exceedingly rare. Further, elasmobranchs rarely experience infections from injuries and appear to recover quickly in the presence of wounds [4–6]. Unlike most vertebrates, elasmobranchs naturally synthesize small single chain antibodies that help counteract a broad range of pathogens [7, 8]. Distinctive elasmobranch compounds are being studied for the treatment of certain cancers, age-related macular degeneration, viral infections, autoimmune diseases, and Parkinson's disease [4]. While studies from other systems confirm that microbiomes exert critical effects on animal immune status and health [9, 10], it remains unknown how the immune properties of elasmobranchs interact with or are shaped by the resident microbiome.

**Fig. 1:**
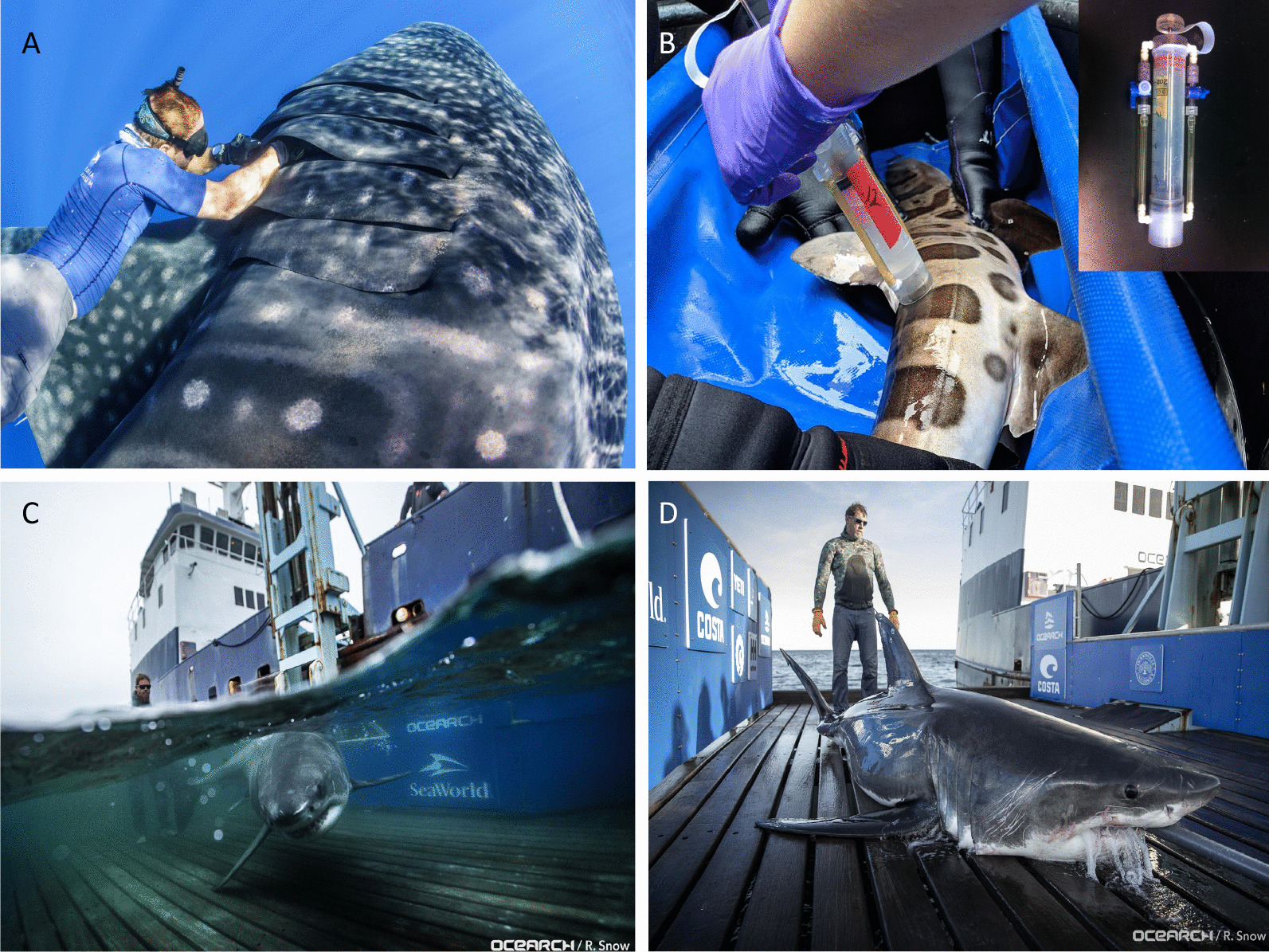
Sampling elasmobranch microbiomes poses physical and technical challenges. Sampling techniques vary among species, locations, and research groups. Microbiome samples have been collected by freediving and swabbing free-swimming animals (**A**) or immobilizing individuals out of water and collecting microbial biomass by swabbing or using custom equipment, such as modified suction devices (**B** with inset). Sampling large pelagic individuals may involve modified vessels equipped with platforms that raise and secure caught individuals (**C**, **D**), providing a unique opportunity to sample species that are hard to capture and restrain. Panel **A** Gill swab from a free-swimming whale shark (Simon Pierce, Marine Megafauna Foundation). Panel **B** Supersucker sampling device (inset: Michael Doane, Flinders University) being used to sample a leopard shark (Elizabeth Dinsdale, Flinders University). Panel **C** White shark on submerged OCEARCH platform (Robert Snow, OCEARCH). Panel **D** White shark on raised OCEACH platform being secured prior to sampling (Robert Snow, OCEARCH)

**Fig. 2:**
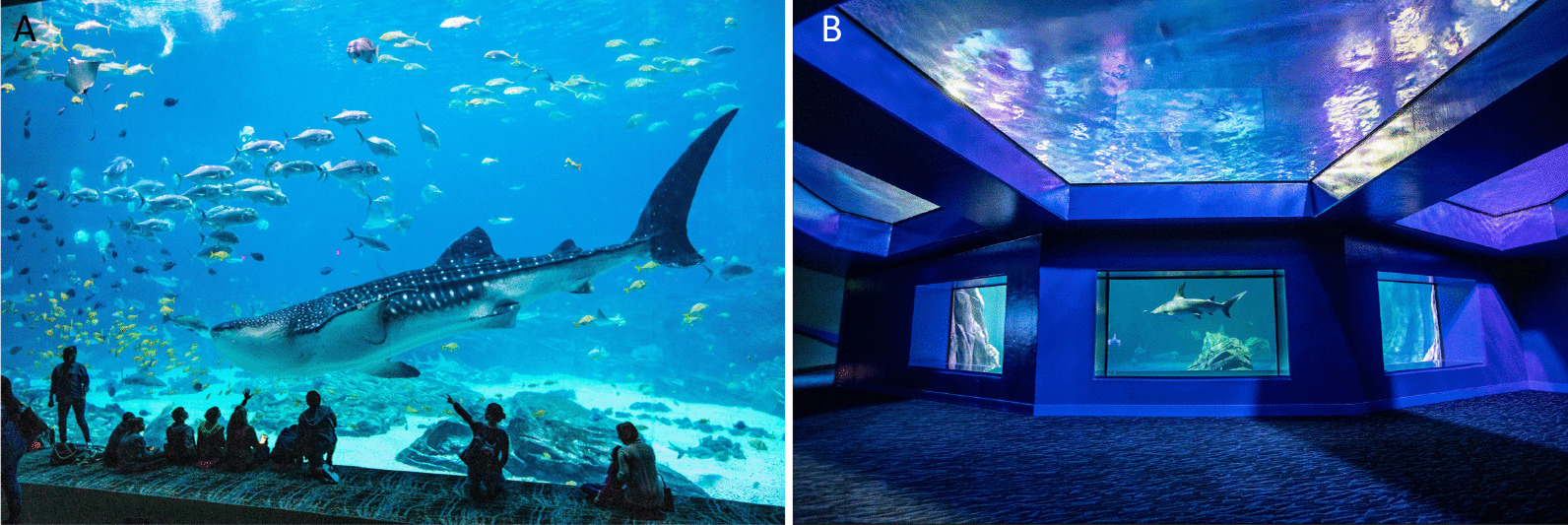
Managed care of elasmobranchs in aquariums provides a unique opportunity for sampling microbiomes over time and relative to monitored host and environmental parameters. Exhibits such as Georgia Aquarium’s Ocean Voyager (**A**) and Sharks: Predators of the Deep (**B**) are enabling studies to understand the drivers of microbiome structure and its role in host health. Panel **A** Whale shark swimming in Georgia Aquarium’s Ocean Voyager exhibit (Chris Duncan, Georgia Aquarium). Panel **B** Hammerhead shark swimming in Georgia Aquarium’s Sharks: Predators of the Deep exhibit (Chris Duncan, Georgia Aquarium)

**Fig. 3:**
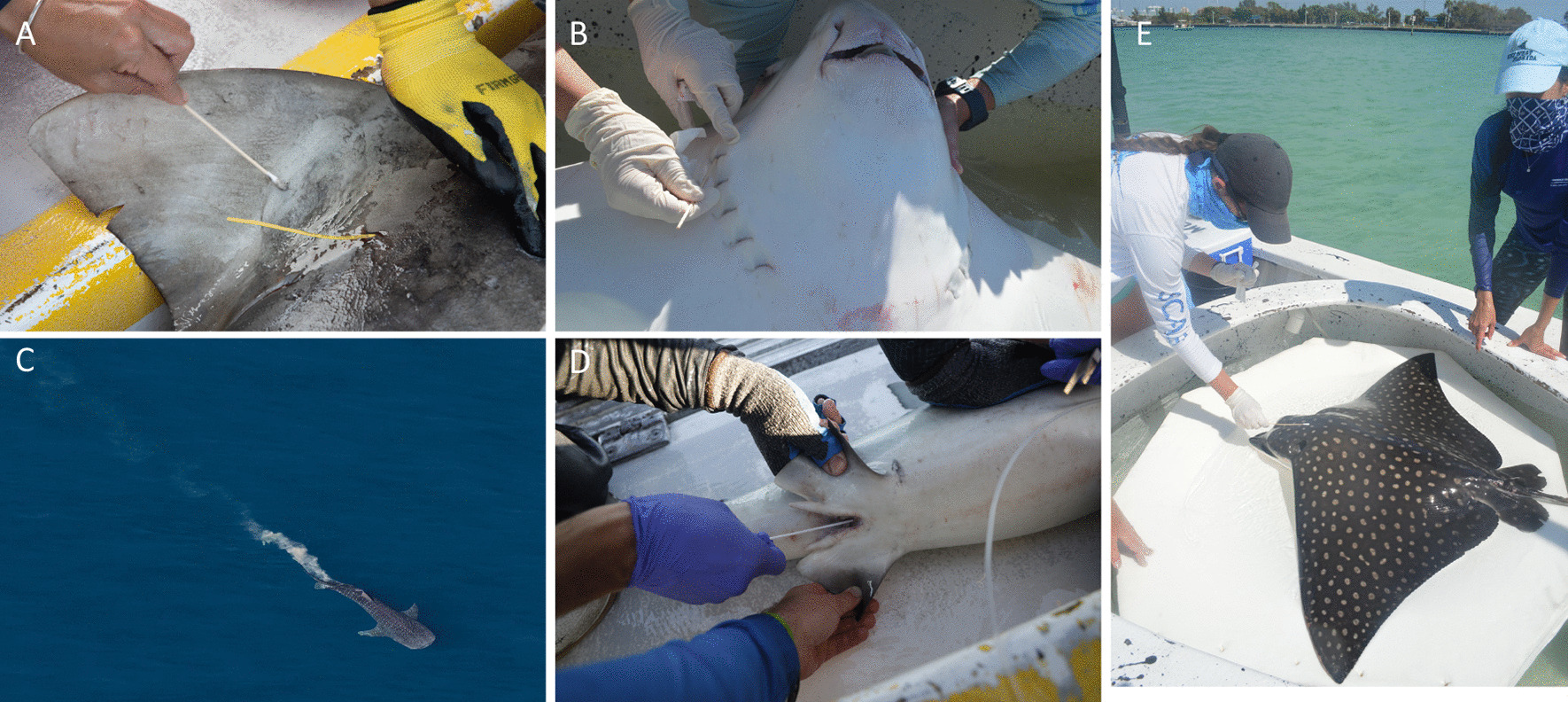
Despite the difficulty of sample collection, elasmobranch microbiomes have been sampled from diverse body niches. Swabbing of the skin/mucus (**A**, **E**) and gill (**B**) is relatively non-invasive and captures microbiomes reflecting both host-specific taxonomic signatures, as well as signatures of the surrounding seawater water microbiome. Host-specific signatures may be driven partly by variation in mucus content and prevalence, such as between sharks and rays. Sampling of gastrointestinal microbiomes has involved opportunistic sampling of feces (**C**) or swabbing of the cloaca (**D**), with cloacal communities representing a transition between external and internal microbiomes. Few studies have examined microbiome variation along the GI tract in dissected individuals. Diet, intestinal anatomy, and host foraging ecology may influence GI microbiome structure. Panel **A** Dorsal skin swab of a tiger shark (Mote Marine Laboratory). Panel **B** Gill swab of a spotted eagle ray (Mote Marine Laboratory). Panel **C** Aerial photograph of a whale shark defecating (Tiffany Klein, Ningaloo Aviation). Panel **D** Cloaca swab of a tiger shark (Mote Marine Laboratory). Panel **E** Dorsal swab of a spotted eagle ray (Mote Marine Laboratory)

Interest in interactions between fish and commensal microbes has increased notably in recent years, although much of this work remains focused on teleost fishes [e.g., 11, 12]. Early work on elasmobranch-associated microbes focused primarily on disease [13] and typically used culture-based approaches to identify a subset of microbial taxa common to elasmobranchs [5, 14–20]. Only recently have DNA sequencing-based studies begun to provide a holistic understanding of elasmobranch microbiology [10, 21]. These and similar studies are facilitated by sustained efforts to find, track, and sample elasmobranchs in the wild, which can be challenging. Specialized vessels or equipment for sampling elasmobranchs safely and humanely, in addition to research on animals under managed care, have allowed for improved access to individuals (Figs. 1, 2, 3). Such work is critical as it informs our understanding of elasmobranch immunity, disease, and the potential for microbe–host relationships to change under environmental disturbance or managed care.

**Fig. 4:**
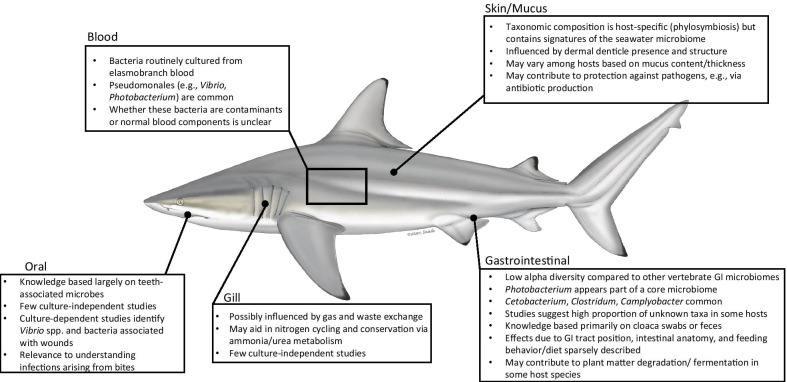
Microbiomes differ among elasmobranch body niches. (Marc Dando, Wildlife Illustrator)

Elasmobranch microbiome research has targeted a small fraction of host species, suggesting that our knowledge of the diversity and function of associated microbes is sparse. We have, for example, a limited understanding of the extent to which microbiome members are shared across hosts and environments and the mechanisms through which microbes interact with the unique physiology of elasmobranchs. To help close this knowledge gap and guide future research, this review summarizes current knowledge of elasmobranch microbiomes based on data from 43 elasmobranch species across 26 studies. Using these important studies as a baseline, we highlight key questions for exploring the roles of microbes in elasmobranch health, physiology, and ecology. We organize the review into subsections covering different niches of elasmobranch anatomy, beginning with the gastrointestinal (GI) niche followed by those of the oral cavity, skin/mucus, and blood (Figs. 3, 4). While microbial pathogenesis in elasmobranchs is not covered in detail in this review, the question of how a commensal elasmobranch microbiome interacts with pathogens is an important target for future research. We direct readers to Garner [22], Borucinska [23], Stidworthy et al. [24], and Stedman and Garner [25] for reviews of elasmobranch pathogens.

## Gastrointestinal microbiomes

Microbes in the vertebrate GI tract affect host digestion, development, immunomodulation, suppression of pathogens, and overall health [26–28]. Knowledge of the diversity and function of GI microbiomes is based primarily on mammals, which account for < 10% of vertebrate diversity [29]. However, GI microbiomes are presumed to play similarly important roles in fishes [12]. As in mammals, GI microbiomes in fishes vary among host species [30, 31], individuals [32], life stages [33], locations in the GI tract [34–36] and in response to seasonal, environmental, or diet variation [37–39]. These patterns, based mainly on observations in teleost fishes, indicate a complex fish microbiome shaped by diverse environmental, physiological, and genetic factors [40, 41]. A similar level of variable community organization is assumed for elasmobranch GI microbiomes, driven at least in part by dynamic environmental and host-associated factors.

As in other fishes [e.g., 42], development mode and environmental exposure likely affect the initial composition of the elasmobranch GI microbiome. The major sources of microbes entering the fish GI tract are the surrounding water, parents, or food [12], with incoming microbes either becoming part of the resident community or passing through as transient members [12, 43]. For example, in a study of yellow stingrays (*Urobatis jamaicensis)* in an aquarium, the microbiome of the cloaca (which contains fecal and GI residues [44]) differed significantly between rays that were wild-caught versus born in the aquarium, suggesting that initial microbial colonization may be driven by environmental parameters [45]. In certain teleost fishes, colonization involves specific bacteria, linked to variations on the egg surface [41]. As in other vertebrates, variation in teleost microbiome composition is greatest earlier in life and then decreases with age [46]. However, far less is known about colonization factors and how the microbiome changes with development in elasmobranchs. Unlike many teleost fishes for which fertilization is external in the water column, elasmobranch fertilization is internal. Internal fertilization is followed by development either in an external egg case or internally with subsequent live birth of offspring. It is therefore possible that initial colonization of the elasmobranch GI system differs, at least partly, from that in teleost fishes, although this remains to be tested.

After colonization, the fish GI microbiome is shaped by a combination of environmental and biological factors, the most important of which may be diet. Elasmobranchs are traditionally classified as carnivores [47], although elasmobranch diets are complex with some species consuming fishes (including other elasmobranchs) or marine mammals, and others feeding on crustaceans or zooplankton. Additionally, elasmobranch diets may shift with age, development, prey availability and environmental conditions. In other vertebrates, diet shifts are tightly linked to microbiome shifts, due primarily to selection for microbes specializing on different nutrient and carbon substrates, but potentially also to the input of microbes attached to food items [48]. For example, GI microbiomes of Atlantic salmon (*Salmo salar*) were shown to change with diet, with the greatest change in microbiome composition associated with a transition to a reduced protein diet [49]. Recently, Leigh et al. [50] explored the gut microbiome of bonnethead sharks (*Sphyrna tiburo*), a species that periodically ingests marine plants. The study found enzymatic evidence of fermentation, a microbial metabolism often, but not exclusively, associated with the degradation of plant material. While it remains uncertain if bonnetheads purposefully graze seaweeds or ingest them incidentally, the finding suggests a role for the microbiome in providing plant-based nutrition to the host and highlights the benefit of using enzymatic characterizations to probe questions of diet–microbiome interaction in elasmobranchs.

The elasmobranch–microbiome relationship may vary depending on the host’s feeding strategy and physiology. Many elasmobranchs consume large meals on an infrequent basis and fast between meals [e.g., 51]. While GI microbiomes shift during fasting in teleost fishes and mammals [40, 43, 52], microbiome change between feedings have not been conducted for elasmobranchs. Gut microbes may play a comparatively substantial digestive role in elasmobranch species that feed infrequently, as the host needs to maximize nutrient extraction to sustain itself until the next meal. By contrast, in elasmobranch species that feed more continuously, the gut microbiome may play a comparatively minor digestive role as food moves quickly through the intestine [53]. Food retention time in some elasmobranchs is controlled in part by a unique anatomical feature, the spiral valve. Present in the lower portion of the intestine in some elasmobranchs, this corkscrew-shaped adaptation increases surface area and slows food passage. Based on data from terrestrial mammals, host species with slower food passage are associated with higher microbiome diversity [54], potentially because increased time for digestion allows for microbial niches associated with degrading chemically challenging compounds [48]. While intestinal anatomy varies among species in both elasmobranchs and teleost fishes [52, 55], the exact relationships among intestinal anatomy, feeding frequency, and microbiome dynamics remain to be determined.

The GI microbiome may be influenced by the host’s nitrogen needs. Elasmobranchs rely on nitrogen for both protein production and osmoregulation, as they use urea (an organic nitrogen compound) as a primary osmolyte [56, 57]. As a consequence, elasmobranchs are likely nitrogen-limited in the wild [58]. The physiology of the elasmobranch valvular intestine may be important for the retention and scavenging of nitrogen. After food consumption, the intestine receives an input of urea, which occurs in the GI chyme, and serves to equilibrate osmotic pressure between the intestine and bodily fluids [59]. The nitrogen in urea is then reabsorbed by the intestines rather than excreted [60]. However, as a metabolic waste product, urea cannot directly be used for making new amino acids. Rather, it is thought that intestinal microbes first convert urea to ammonia, which can then be incorporated into amino acids [61]. Wood et al. [61] observed activity of urea-degrading enzymes (ureases) in the intestines of the Pacific spiny dogfish (S*qualus suckleyi)* and another chondrichthyan, the Pacific spotted ratfish (*Hydrolagus colliei*), at a level higher than in ammonia-excreting teleost fish. Ammonia scavenging via urease-expressing gut microbes has been observed in other animals (e.g., during hibernation; [62, 63]). The extent to which this process influences microbiome composition and occurs across elasmobranch species is unknown.

Despite their potential for variation, elasmobranch GI microbiomes share certain broad patterns of composition and diversity (Fig. 4; Additional file 1: Table S1). Sherrill-Mix et al. [28] showed that elasmobranch microbiomes, albeit from a handful of host species, cluster apart from those of other animals based on microbial taxonomic composition, exhibit relatively low diversity, and are typically dominated by a small number of operational taxonomic units (OTUs). Diversity levels in elasmobranch GI microbiomes are more similar to those of insect microbiomes than to those of other vertebrates (although teleost fishes were not well represented [28]). Similarly, in a study of three shark species (*Carcharhinus plumbeus*, *Carcharhinus brevipinna*, *Rhizoprionodon terraenovae*), Givens et al. [26] showed that shark intestinal microbiomes have low species richness and phylogenetic diversity. This pattern was also observed in juvenile scalloped hammerheads (*Sphyrna lewini*), in which two microbial OTUs (assigned to *Citrobacter koseri* and *Photobacterium damselae*) dominated the microbiome and were detected in all individuals [64]. However, sampling of a broader range of host taxa is needed to confirm if low diversity is a general feature of elasmobranch gut microbiomes. Such a pattern may indicate either a sparsity of biochemical niches in the gut, a relatively strong environmental filtering that restricts the community to only a few members, or that elasmobranchs actively regulate the communities in their gut.

Whether certain microbial taxa or biochemical functions are conserved across all elasmobranchs remains to be determined. Givens et al. [26] found that microbiomes shared 69–98% of OTUs among individuals of the same shark species. However, only three OTUs were shared among the three shark species; these included OTUs assigned to the bacterial genera *Cetobacterium*, *Vibrio*, and *Photobacterium. Vibrio* and *Photobacterium* are Gammaproteobacteria in the Family Vibrionaceae and are particularly common in elasmobranchs. *Photobacterium* spp. was the most abundant OTU in at least six elasmobranch species [26, 28, 64]. A dominance of *Photobacterium* has also been reported in teleost fishes [26, 30, 43, 65]. *Photobacterium* and *Vibrio* presumably share metabolic traits, such as the production of hydrolytic enzymes and breakdown of host dietary components [12]. Urease activity was detected in shark skin-associated strains of these genera [17, 56], raising the possibility that related GI strains may play a role in urea breakdown and nitrogen retention. Conversely, both *Photobacterium* and *Vibrio* are associated with fish diseases [66]. *Vibrio alginolyticus*, for example, is sometimes pathogenic, but also works as a probiotic, protecting Atlantic salmon from pathogens, including other *Vibrio* species [12, 67]. Similarly, while *Photobacterium damselae* is a pathogen of wild and captive teleost fish [66, 68], other *Photobacterium* may be mutualistic, for example by aiding in chitin digestion [12]. The emerging data from sharks, although representing a small number of species, suggest that *Photobacterium* and *Vibrio* play important roles in the elasmobranch intestine, although their specific contributions to elasmobranch health and nutrition remain to be ascertained.

Other taxa common to elasmobranch GI microbiomes include bacteria of the Firmicutes (e.g., *Clostridium* spp.), Fusobacteria (e.g., *Cetobacterium* spp.) and Actinobacteria [26, 28, 50, 64]). These lineages are ubiquitous in the guts of teleost fish [e.g., 12, 69], although their abundances can vary substantially among individuals and species. Notably, *Clostridium* has been detected in nearly all individuals across all shark species, representing 0.01 to 37% of sequences [28, 64]. This Gram-positive genus includes both pathogenic and mutualistic members. While the physiology of most fish-associated *Clostridium* lineages is not verified, isolates from teleost fishes suggest diverse functions, including protein degradation, fermentation and fatty acid production, antimicrobial activity, and host immune system priming [12, 70–72]. Prior work from other systems suggests potentially diverse physiological contributions by the other microbial groups common to the elasmobranch gut. For example, in freshwater fish, *Cetobacterium* is associated with cellulose degradation and the synthesis of vitamin B-12 for the host [73, 74], while diverse *Actinobacteria* isolates exhibit antimicrobial activity [75]. *Actinobacteria* produce secondary metabolites and, in mammalian systems, have been implicated in the regulation of anti-inflammatory cytokines [76].

Genomic and culture-based analysis, as well as additional taxonomic profiling, are needed to determine the role of these and other microbes in the elasmobranch GI system. Such studies would benefit from sampling across host species, diet/feeding strategies, and developmental stages to identify factors that covary with microbiome composition. Studies should also explore how microbiomes vary along the GI tract. An early culture-based study recovered diverse *Vibrio*, *Photobacterium*, and *Alteromonas* OTUs from different GI sites (esophagus, stomach, pylorus, duodenum, and spiral intestine) in two shark species [18], raising the possibility of microbe–host interactions at sites other than the intestine. Indeed, characterizations of the elasmobranch GI microbiome often are based on swabs of the cloaca or opportunistic collections of feces. Both methods have the potential to also sample non-GI microbiomes and to represent only a fraction of the microbial diversity along the GI tract.

## Oral microbiomes

The oral microbiome of vertebrates has long been of interest as an interface between internal and external microbiomes, for its role in diseases of the oral cavity and other systemic ailments, and as a model for understanding microbe–microbe interactions [77, 78]. A handful of studies have begun to explore the oral microbiome of elasmobranchs, driven partly by the potential (though seemingly remote) that human infections could arise from shark bites.

**Fig. 5:**
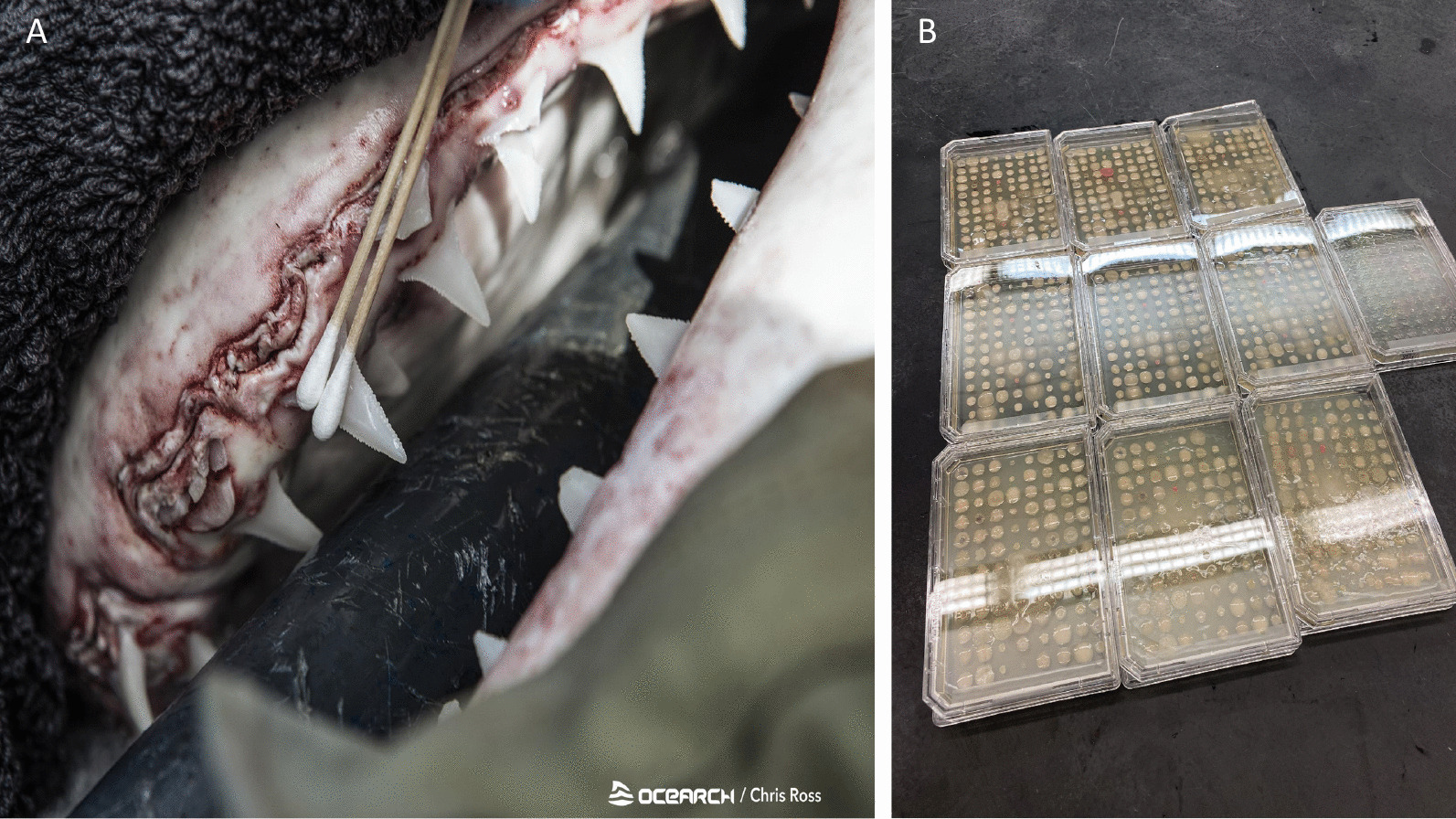
The elasmobranch tooth microbiome (**A**) has been of interest for medical treatment of elasmobranch bites on human swimmers, although the incidence of negative elasmobranch–human interactions is rare. Culture-based exploration (**B**) is an avenue for future research to examine biochemical and ecological aspects of elasmobranch-associated microbes. Panel **A** Oral swab being collected from a secured white shark (Chris Ross, OCEARCH). Panel **B** Culture plates from elasmobranch oral swabs (Kim Ritchie, University of South Carolina Beaufort)

Most studies of the elasmobranch oral microbiome have focused on microbes isolated from teeth swabs (Figs. 4, 5; Additional file 1: Table S2). In a culture-based study, Buck et al. [15] isolated 24 strains, primarily *Vibrio* sp., from the teeth of a white shark (*Carcharodon carcharias*). Similarly, Interaminense et al. [19] investigated the oral cavity (teeth and under the gums) of bull (*Carcharhinus leucas*) and tiger (*Galeocerdo cuvier*) sharks using culturing and identification via biochemical profiling. They reported a high incidence of members of the gammaproteobacterial Family *Enterobacteriaceae*, which includes genera cultured from wounds (e.g., *Klebsiella*, *Citrobacter*), and taxa often associated with human presence (e.g., *Escherichia coli*); the authors speculated that this trend was related to low water quality at the sampling sites. Another culture-based study found similar results, recovering common Gram-negative (*Vibrio* and *Pasteurella* sp.) and Gram-positive (*Staphylococcus* and *Bacillus* sp.) representatives from the mouths of blacktip sharks (*Carcharhinus limbatus*; [20]). However, culture-based analyses, though valuable, do not accurately measure community taxonomic composition. A recent non-culture-based study found that teeth microbial communities differed in diversity, richness, and composition among five shark species, suggesting that species-specific differences were driven by variations in diet and feeding behavior [79]. Despite recent progress, the diversity and function of microbes in the elasmobranch mouth remain largely uncharacterized.

A potential linkage between oral microbes and bite-associated infections in humans also remains unclear. Several studies have cultured bacteria from the wounds of shark bite victims. The isolated bacteria include taxa commonly associated with animal disease, including species of *Vibrio, Aeromonas*, *Klebsiella*, *Citrobacter*, and *Enterococcus* [80–82]. However, many of these taxa, notably *Vibrio* species, are ubiquitous in the marine environment, including on elasmobranch teeth (see above). It is therefore difficult to know if their presence in bite wounds is due to entry from the water column or from the shark’s oral microbiome. The same challenge exists for human infections arising from contact with stingray barbs. Additional studies of oral and barb-associated microbiomes could inform knowledge of the origin of infectious agents (teeth/barb vs. seawater), as well as inform medical care and wound management [83, 84].

## Skin/mucus-associated microbes

Much elasmobranch microbiome research has focused on the skin and its associated mucus layer, although knowledge of these external communities remains sparse for elasmobranchs compared to other vertebrates [85]. The integumentary system (skin) of elasmobranchs is one of the largest organs in the body, is biologically active, and lacks a keratinized outer surface as in human and terrestrial animals [86]. The outer skin is composed of the epidermis tissue and associated mucus and in aquatic organisms plays key roles in osmoregulation [87], chemical communication, social behavior [88], and protection from abrasion [87], toxins [89], and pathogens [90, 91]. Indeed, diverse microbiomes have been detected on the skin of elasmobranch and teleost fishes, primarily by culturing or 16S rRNA gene analysis of microbes sampled by swabbing the skin surface (Figs. 3, 4; Additional file 1: Table S3). Compared to microbiomes internal to the body, the fish skin microbiome likely has high connectivity with that of the surrounding environment. However, skin microbiomes may also have host-specific signatures driven by variation in skin and mucus properties.

Elasmobranch skin/mucus differ among species and compared to those of teleost fish. While teleost skin generally has scales, elasmobranch skin is covered with toothlike projections called dermal denticles. Also known as placoid scales, dermal denticles act as body armor and reduce drag during swimming [86, 92]. In most sharks, dermal denticles cover the entire body but vary in size and structure among species with different swimming modes (e.g., slow vs. fast; [93]). In contrast, demersal elasmobranchs, notably certain batoid species, have few denticles or lack them completely [84]. While teleost skin has a relatively thick mucus layer, elasmobranch skin exhibits wide variation in mucus layer thickness, from inconspicuous in some shark species to relatively thick in skates and batoids.

The variable and complex nature of elasmobranch skin and mucus almost certainly impacts the skin microbiome. By altering the hydrodynamic properties of water flow close to the epidermis [94], denticles create a flow boundary that minimizes settlement of microbial cells on the skin surface [95, 96]. However, the ridges and microstructures of denticles encourage settlement of cells that break through the flow boundary. Studies using fabricated surfaces patterned after shark skin show that denticle-like structures promote the initial attachment of bacteria but inhibit development of thick biofilms [96–98]. The relatively thin mucus layer on shark skin may also limit microbial biomass. While mucus can facilitate microbial adhesion and provide a protective matrix and nutrients for microbial growth [99], it can also be antagonistic to microbes. Fish mucus chemistry, although focused primarily on teleosts, reveal diverse antimicrobial molecules, including lysozyme and proteases [100, 101]. While the chemistry of elasmobranch mucus is more sparsely characterized, studies of skin mucus from two species have also identified antimicrobial compounds, including a C-type lectin in mucus of the Japanese bullhead shark (*Heterodontus japonicas*) and pentraxin, an antimicrobial pectin from mucus of the common skate *(Raja kenojei*) [102, 103]. While some antimicrobial molecules in mucus are host-derived [104], some may be produced by skin-associated microbes and, as in other microbial biofilms, likely play a role in structuring the composition of the skin microbiome.

Factors other than mucus cover also play a role in structuring the skin microbiome. Environmental conditions of the water and the taxonomic identity of the host may affect microbial community structure. For example, the taxonomic composition of bacteria isolated from Atlantic stingrays *(Hypanus sabinus*) was shown to differ between animals obtained from fresh versus marine waters [5]. Similarly, microbiome composition on blacktip reef sharks (*Carcharhinus melanopterus*) differed significantly among individuals collected from different sites within the Seychelles [105]. However, despite differences in geographic patterns, elasmobranch skin microbiomes are compositionally distinct from those of the surrounding water [9, 79], confirming the skin as a distinct host-associated niche. Indeed, skin microbiome composition varies among host taxa and, in some instances, host-specific variation may be greater than that due to environmental or geographic differences. Recent evidence suggests that microbiome composition diverges in parallel with host identity and that this ‘phylosymbiosis’ signal is stronger than that in teleost fishes [105]. Further sampling across the elasmobranch phylogeny and over environmental and seasonal gradients is needed to resolve the extent and magnitude of phylosymbiosis in elasmobranchs and potentially identify the factors (e.g., variation in skin/mucus properties) underlying host-specific patterns.

Only a handful of studies have explored the metabolic activities of elasmobranch skin microbiomes or confirmed their role in host health. In one of the only metagenomic assessments of an elasmobranch skin microbiome, Doane et al. [9] compared the skin microbiome of the common thresher (*Alopias vulpinus*) to that of the surrounding water and found that the microbiome was enriched in genes for social interactions (e.g., virulence), iron acquisition, and heavy-metal tolerance. Selection for social interactions is anticipated for microbes living in physical association with neighbors in a skin biofilm. Doane et al. [9] hypothesize that functions related to resource (e.g., iron) acquisition may also be due to competition among biofilm-associated cells, whereas enrichment of genes for metal tolerance is related to the tendency for sharks to accumulate heavy metals in their tissues. A follow-up study examining diverse fish species showed functional gene differences between elasmobranch versus teleost skin microbiomes [106]. The analysis identified trends potentially driven by differences in the availability and use of mucus, with teleost microbiomes enriched for functions of mucus component degradation (e.g., protein metabolism) and elasmobranch microbiomes enriched for functions of attachment and biofilm formation (e.g., motility, chemotaxis).

Elasmobranch skin microbiomes, similar to teleost skin microbiomes [e.g., 107], may be enriched in virulence or antimicrobial properties. For example, Ritchie et al. [5] isolated 1,860 bacteria from the epidermal mucus of rays and skates, detecting antibiotic activity in 17% of the isolates, a proportion similar to that observed among isolates from coral mucus [84, 108]. Bacteria with antibiotic-production potential have since been detected on the skin of both wild rays and those under managed care [5, 84, 109]. These bacteria represent Gram-positive and -negative groups with members known from other studies to produce broad-spectrum antibiotics (Additional file 1: Table S3). These results raise the prospect that elasmobranch skin-associated microbes may help fight infection.

Although this hypothesis has yet to be experimentally verified, there is evidence that elasmobranchs are relatively resistant to infections, at least infections associated with lacerations. Like all animals, elasmobranchs are susceptible to injury through encounters with other animals and, increasingly, humans [e.g., 110]. Several reports have claimed that elasmobranchs, particularly sharks, recover from injuries rapidly and without infection [e.g., 111–115]. However, healing rates have been measured for only a small number of species and are variable among taxa [e.g., 115, 116], making it hard to determine a baseline healing rate for comparison. Despite this ambiguity, elasmobranchs appear capable of recovering from major injuries, including lacerations that involve significant resource allocation or physiological/behavioural adjustments [115, 117]. Only recently have studies begun to explore the role of the skin microbiome in wound recovery. Pogoreutz et al. [105], for example, examined microbiomes associated with the skin covering the gills and back of injured blacktip reef sharks in the Seychelles, but did not detect microbial taxonomic differences between visibly healthy versus injured skin. Nonetheless, such results do not rule out the potential that the skin microbiome may deter opportunistic pathogens. It is also uncertain if a protective benefit of the skin microbiome might vary between species with versus without a mucus coat.

It is unknown how or if the elasmobranch skin microbiome varies in comparison to other exterior body sites, notably the gills. Elasmobranch gills may be a unique habitat for microbes given the role of the gills in waste excretion, gas exchange, and possibly ammonia recycling. Elasmobranchs excrete nitrogen from the gills in the form of urea [60]. Remarkably, elasmobranchs also have the unique capacity to absorb ammonia through the gills, potentially to help replenish urea used for osmoregulation [118, 119]. The mechanism behind branchial ammonia uptake is not fully understood [118], nor is the role of urea and ammonia exchange in shaping a gill-associated microbiome. Some studies have isolated and detected *Vibrio* species from elasmobranch gills [16, 17, 45]. Many *Vibrios* exhibit urease activity, raising the hypothesis that urea exchange may influence the gill microbiome and, conversely, that microbiome urease activity may contribute to ammonia production on the gills [61]. Focusing on teleost fishes, Pratte et al. [30] found that the gill microbiome is distinct from other external body sites (skin). Similar culture-independent studies, for example using metagenomics, could help identify metabolic functions enriched in gill-associated microbes compared to those from skin sites less influenced by host nitrogen cycling.

## Blood-associated microbes

For vertebrates, it is assumed that having bacteria in the blood is linked to negative health outcomes. While bacteria may enter the blood of healthy individuals, these events are short-lived if the immune system is not compromised. If the immune system is overwhelmed, proliferating bacteria can result in sepsis, a life-threatening organ dysfunction caused by aberrant host response to infection. In contrast to this assumption, bacteria have been cultured repeatedly from the blood of healthy elasmobranchs (Fig. 4; Additional file 1: Table S4). These include Gram-positive and -negative heterotrophs commonly recovered from both planktonic and host-associated marine microbiomes, notably genera of the ubiquitous order Pseudomonales (e.g., *Vibrio, Photobacterium, Aeromonas, Moraxella*; [16, 18]). A study of 195 individuals representing 12 species recovered culturable bacteria from 21% of sharks and 50% of rays, noting that cultures were more often recovered from pelagic species (38.7%) compared to sedentary species (18.3%) [3]. However, the authors acknowledge that some samples may have been contaminated from needle passage through muscle or skin tissue. Tao et al. [120] also isolated bacteria, primarily *Vibrio* species, from blood of the lesser electric ray (*Narcine bancroftii*), with many of these isolates being distinct from reference strains and potentially representing new species of *Vibrio*, *Amphritea*, *Shewanella*, and *Tenacibaculum*. As many of these genera are also found in marine sediments, the authors posited that microbes may enter the host by ingestion of sediments during benthic feeding. If so, the bacteria would then enter the bloodstream, presumably via entry across the intestinal lining.

The repeated detection of bacteria in elasmobranch blood suggests that non-sterile blood is a baseline condition in this major aquatic group, challenging the classical assumption that bacteria in blood indicates disease. Elasmobranchs are an ancient vertebrate lineage and one of the first to evolve adaptive immunity [121], and therefore, sharks have long been important targets for immunology research [122]. Their immune systems share important properties with those of humans, while also showing key differences, including the presence of rare single chain antibodies [123]. Further, sharks rarely experience infections [4]. If these and other unique immune properties explain, or can be explained by, the persistence of microbes in the blood (outside of a disease state), then characterizing these microbes may have implications for understanding why immune systems evolved differently among vertebrate groups. However, additional work is needed to confirm that bacteria persist as metabolically active ‘residents’ in elasmobranch blood.

## Conclusions

Elasmobranch microbiome research has intensified dramatically in recent years. This work has been motivated in part by a need to better understand the health of rays and sharks as these ecologically important animals continue to face significant environmental and anthropogenic stressors [124]. Additionally, understanding of baselines in the microbiome community will allow best care practices for elasmobranch in managed care facilities. Further, the unique physiology of elasmobranchs pertaining to metabolism, osmoregulation, and immunity suggests the potential that elasmobranch–microbe interactions are distinct from those in other vertebrates, including teleost fishes. In cases where poor host health may involve a microbial component—either a specific pathogen or an imbalance in the microbiome (dysbiosis)—it may be unclear if negative health effects are due to resident microbes that changed from commensal to harmful as conditions changed, colonization by outside pathogens, or both. Distinguishing among these processes is a priority but requires a clearer understanding of which microorganisms do or do not constitute health threats in elasmobranchs, as well as studies that assess the microbiome over changes in host health, e.g., due to stress, disease, or wounding and recovery. Such studies remain rare for elasmobranchs, potentially due in part to the relative novelty of considering disease in the context of microbe–microbe interactions [21], but likely also to the challenges of working with these animals.

Sampling elasmobranch microbiomes can be difficult. Not only are many elasmobranchs challenging to capture, but substantial resources are also required to obtain the sample size necessary for statistical analysis. Capturing elasmobranchs can require specialized vessels and equipment to minimize risk to the animals and the researchers. Once captured, live animals must be handled with care and usually only for short periods of time to avoid stressing or injuring the animal. Microbiome sampling may therefore be restricted to quick, non-invasive swabs of the skin or other external surfaces. Elasmobranch fecal samples may be collected only opportunistically and are particularly rare for large migratory or deep-sea species. Fortunately, the potential for collecting data on large elasmobranchs is increasing. This is due in part to the work of organizations such as OCEARCH [125] that provide expertise and resources for sampling large animals safely and humanely. Such work can coordinate diverse sampling goals, allowing microbiome data to be coupled to host and environmental parameters. Elasmobranchs caught in fisheries can also be sampled for microbiome analysis. However, the potential for microbiomes to change rapidly after death could bias data from fisheries-captured elasmobranchs. Access to live specimens is therefore vital, as is ensuring that organisms are captured and released safely and humanely. Ideally, microbiome sampling of live animals should be paired with sampling of host physiology (e.g., fatty acid profiles, heavy metal concentrations, oxygen consumption, or reproduction status) to establish the role of the microbiome in host health.

Keeping individuals under managed care creates opportunities for experimentation and microbiome sampling over time. The latter is valuable for assessing microbiome stability and would ideally be coupled with measurements of host physiology and environmental conditions, including characterizations of the seawater microbiome. Holistic datasets of this sort would allow researchers to distinguish residents from transient microbiome members, quantify the degree to which the microbiome is affected by environmental and host factors (eg., diet shifts, disease), and identify those microbial taxa most relevant to host health. Though valuable, studies of individuals under managed care present challenges. Notably, many elasmobranchs, particularly larger species, can be hard to house in aquaria. There also is no guarantee that conclusions drawn from these animals apply to those in the wild. Despite these caveats, academic and commercial aquariums have had long term success in maintaining healthy elasmobranchs. These institutions often maintain detailed animal health and diet records and may engage in conservation and veterinary research that could easily integrate a microbiome component. Standardization of microbiome sampling methods across institutions could be relatively straightforward and would enable comparisons across diverse aquaria-housed species, environmental conditions, and potential changes in host disease state. Collecting microbiome samples from aquaria-housed elasmobranchs is relatively non-invasive and inexpensive and should be considered in monitoring and time-series research plans to understand host health.

Elasmobranch microbiomes have thus far been understood primarily through marker gene surveys targeting the phylogenetically informative 16S rRNA gene. These surveys provide valuable insight into community taxonomic diversity. However, these surveys only infer, but do not confirm, the ecological roles of microbiome members based on the assumption that a microbe’s function is aligned with its phylogenetic placement. However, horizontal gene transfer, genomic scavenging, and phage infection can change the ecological role of a microbial strain [126]. Shotgun sequencing of community DNA (metagenomics) characterizes both taxonomically informative marker genes and protein-coding metabolic genes and thereby provides insight into the ecological potential of a microbiome. While this method is widely used in microbiome research in general (e.g., [127]), it has thus far been applied in a small number of elasmobranch microbiome studies. These studies have revealed microbiome-host co-diversification [106], metabolic functions enriched in elasmobranch microbiomes [9], and a large proportion of microbiome protein-coding sequences without clear homologs in databases [9]. Future work to more precisely identify the phylogenetic and functional diversity of these sequences may benefit from assembling individual genomic units from metagenome datasets (Metagenome-Assembled Genomes (MAGs); [128]). Such studies have the potential to also provide insight into the host’s genomics. For example, shotgun sequencing of community DNA from the skin of the common thresher allowed reconstruction of the host mitochondrial genome, helping to clarify the position of this species in the elasmobranch phylogeny [129]. Metagenomic analysis can also characterize other microbiome members, potentially including fungi, other small eukaryotic organisms, and viruses. Viruses/phage are of particular interest given their role in other systems as modulators of host cell metabolism [130] and drivers of bacterial diversity [131] through processes such as classical predatory–prey relationships [132], but have yet to be characterized in elasmobranch microbiomes.

Future elasmobranch microbiome studies, focused on both wild individuals and those under managed care, should continue to measure community taxonomic composition (16S rRNA gene analysis) but also apply metagenomics and other steps to identify the ecological importance of microbiomes from different body niches. For the intestinal microbiome, metagenome sequencing coupled with metabolomic and diet analysis could identify microbial enzymes or metabolites with roles in host nutrition and energy provisioning, waste or osmolyte processing (e.g., urea/nitrogen cycling), and signaling to the host immune system. The natural variation in diet and feeding strategy (e.g., feasting and fasting vs. grazing) in elasmobranchs creates opportunities to test how such factors influence (or are influenced by) the gut microbiome. Similar analysis of the skin microbiome, potentially comparing wounded versus non-wounded tissue, could be used to test if commensal microbes contribute to the low incidence of wound infection in elasmobranchs, potentially via the production of antimicrobial compounds. Additionally, emerging techniques such as CLASI-FISH (combinatorial labelling and spectral imaging—fluorescence in situ hybridization) can be used to visualize the spatial organization of microbial taxa in biofilms and therefore help identify microbe–microbe interactions in the mucus layer of elasmobranch skin [133]. Other visualization techniques such as scanning electron microscopy can also provide critical insight into how microbes interact physically with elasmobranchs, such as showing how dermal denticle structure influences the colonization and arrangement of bacteria in the mucus layer. Finally, the potential for a blood microbiome in healthy elasmobranchs remains intriguing, but thus far unconfirmed. Prior to investigating the biochemical importance of a blood microbiome, additional studies are necessary to show unequivocally that microbes detected in or cultured from blood are not contaminants and are present at higher frequencies than in other aquatic vertebrates sampled using the same methods. If this can be shown, follow-up questions should explore how these microorganisms interact with host physiology to avoid a strong immune response.

We hypothesize that the unique physiology and behavior of elasmobranchs supports novel microbe–host interactions. Recently, for example, the biofluorescent properties of swell sharks (*Cephaloscyllium ventriosum*) and chain catsharks (*Scyliorhinus retifer*) have been linked to unique brominated tryptophan–kynurenine metabolites, which have antimicrobial properties [134]. Whether and how such adaptations affect (or are affected by) the microbiome remains to be tested. The rapidly advancing pace of elasmobranch microbiome research suggests exciting discoveries in the next decade. Future exploration of these unique microbial ecosystems may identify novel microbial taxa, compounds (e.g., antibiotics), or mechanisms of microbe–immune system crosstalk, as well as inform questions at the interface of elasmobranch–microbe–human interaction (e.g., treatment protocols for shark bite and stingray barb victims, strategies for managed care). Such research has the potential to establish elasmobranchs as important models for animal microbiome science.

## Supplementary Information

**Additional file 1: Table S1.** Gut-associated microbes; **Table S2.** Oral-associated microbes; **Table S3.** Skin/Mucus/External-associated microbes; **Table S4.** Internal tissue-associated microbes.

## Data Availability

Not applicable to this article as no datasets were generated or analyzed during the current study.
