# Supplementary material for: Elasmobranch microbiomes: emerging patterns and implications for host health and ecology
Source: Anim Microbiome. 2021 Sep 15;3:61. doi: 10.1186/s42523-021-00121-4 (PMC8444439; doi:10.1186/s42523-021-00121-4)
Supplement: Supplementary file 1 — Additional file 1: Table S1. Gut-associated microbes; Table S2. Oral-associated microbes; Table S3. Skin/Mucus/External-associated microbes; Table S4. Internal tissue-associated microbes. [file 42523_2021_121_MOESM1_ESM.docx]

**Supplemental Material**

**Elasmobranch microbiomes:**

**emerging patterns and implications for host health and ecology**

Cameron T. Perry^1^, Zoe A. Pratte^1,2^, Ana Clavere-Graciette^1^, Kim B. Ritchie^3^, Robert E. Hueter^4,5^, Alisa L. Newton^6^, G. Christopher Fischer^5,7^, Elizabeth A. Dinsdale^8^, Michael P. Doane^8^, Krystan A. Wilkinson^4,9^, Kim Bassos-Hull^4^, Kady Lyons^10^, Alistair D.M. Dove^10^, Lisa A. Hoopes^10^, Frank J. Stewart^1,2^

*^1^School of Biological Sciences, Georgia Institute of Technology, Atlanta, GA, USA*

*^2^Department of Microbiology & Immunology, Montana State University, Bozeman, MT, USA*

*^3^Department of Natural Sciences, University of South Carolina Beaufort, Beaufort, SC, USA*

*^4^Sharks and Rays Conservation Research Program, Mote Marine Laboratory, Sarasota, FL, USA*

*^5^OCEARCH, Park City, UT, USA*

*^6^Disney’s Animals, Science and Environment, Orlando, FL, USA*

*^7^Marine Science Research Institute, Jacksonville University, Jacksonville, FL, USA*

*^8^College of Science and Engineering, Flinders University, Bedford Park, South Australia,, Australia*

*^9^ Chicago Zoological Society’s Sarasota Dolphin Research Program ℅ Mote Marine Laboratory, Sarasota, FL, USA*

*^10^Research & Conservation Department, Georgia Aquarium, Atlanta, GA, USA*

**Supplemental Table 1: Gut-associated microbes**

| Species | Method | Finding | Source |
| --- | --- | --- | --- |
| Black dogfish (*Centroscyllium fabricii*) | Illumina Miseq sequencing of the V3 region of the 16S rRNA | Actinobacteria (27.84%), Proteobacteria (18.99 %), and Acidobacteria (10.89%) dominated. | **Johny et al. (2018)** |
|  |  | Large portion (25.3%) of unknown OTUs suggesting novel microbes. |  |
| Blacktip shark (*Carcharhinus limbatus*) | Culture | *Vibrio* sp. cultured from the stomach. | **Grimes et al. (1985)** |
|  | Culture | *Photobacterium* *damselae*, *Vibrio* *alginolyticus*, *Vibrio* *harveyi* and *Alteromonas* sp. detected in the gastrointestinal tract. | **Grimes et al. (1993)** |
| Bonnethead (*Sphyrna tiburo*) | V4-V5 region of 16S rDNA | No significant differences in alpha and beta microbial diversity between laboratory fed and wild caught sharks for all gut regions. | **Leigh et al. (2021)** |
|  |  | The proximal intestine had a distinct community structure when compared to the distal and spiral intestine. An unknown Clostridaceae was the main driver of differences. |  |
|  |  | *Vibrionales* accounted for 44.3% to 99.3% of all reads. Top five OTUs were *Photobacterium damselae,* Clostridaceae*,* Peptostreptococcaceae*, Pseudomonas veronii,* and *Photobacterium.* |  |
| Bull shark (*Carcharhinus leucas*) | V1-V2 region of 16S rRNA | *Photobacterium* sp. (0.1-55.9 %), *Clostridium* sp. (0.1-37.3 %), and *Campylobacter* sp. (0.5-37.3 %) were the most abundant taxa from five individuals. | **Sherrill-Mix et al. (2018)** |
| Lemon shark (*Negaprion brevirostris*) | Culture | *Vibrio* *damselae*, *Vibrio* sp., and *Vibrio* *harveyi* cultured from gastrointestinal tract. | **Grimes et al. (1985)** |
|  | Culture | *Photobacterium* *damselae*,*Vibrio* *alginolyticus*, and *Vibrio* *furnissii* detected in gastrointestinal tract. | **Grimes et al. (1993)** |
|  | V1-V2 region of 16S rRNA | *Photobacterium* sp. (89.4 %) was the most abundant taxa from one individual. | **Sherrill-Mix et al. (2018)** |
| Nurse shark (*Ginglymostoma cirratum*) | Culture | *Photobacterium* sp., and *Vibrio* *alginolyticus* cultured from oral cavity. | **Grimes et al. (1985)** |
|  | Culture | *Vibrio alginolyticus* found in the intestinal tract. | **Buck (1990)** |
| Sandbar shark (*Carcharhinus plumbeus*) | V1-V2 region of 16S rRNA | *Photobacterium* sp. (7.1-64.5 %) and unknown taxa (6.6-58.2 %) were the most abundant taxa from five individuals. | **Sherrill-Mix et al. (2018)** |
|  | Pyro and Sanger sequencing of the V1-V9 region of 16S rRNA | Top five most abundant OTUs were *Photobacterium* sp. (2), *Vibrio* sp. 1, *Cetobacterium* sp., and *Vibrio* sp. 2 | **Givens et al. (2015)** |
| Scalloped hammerhead (*Sphyrna lewini*) (juvenile) | Illumina MiSeq sequencing of the V5-V8 region of 16S rRNA | *Citrobacter* *koseri* and *Photobacterium* *damselae* were the two most common OTUs | **Juste-Ponipanen et al. (2019)** |
|  |  | Significant variation between bacterial compositions of individuals. *Citrobacter* *koseri* ranged from 0.11-50.7% in individuals |  |
| Atlantic sharpnose shark (*Rhizoprionodon terraenovae*) | Culture | *Vibrio* *alginolyticus* and *Escherichia* *coli* cultured from gastrointestinal tract. | **Grimes et al. (1985)** |
|  | Pyro and Sanger sequencing of the V1-V9 region of 16S rRNA | Top five most abundant OTUs were *Photobacterium* sp. (2), *Vibrio* sp., *Campylobacter* sp., and *Propionibacterium* sp. | **Givens et al. (2015)** |
| Spinner shark (*Carcharhinus brevipinna*) | Pyro and Sanger sequencing of the V1-V9 region of 16S rRNA | Top five most abundant OTUs were *Photobacterium* sp., *Propiongenium* sp., Clostridiaceae, *Clostridium* sp., and *Vibrio* sp. | **Givens et al. (2015)** |
| Tiger shark (*Galeocerdo cuvier*) | V1-V2 region of 16S rRNA | *Campylobacter* sp. (0.1-54.4 %), *Photobacterium* sp. (4.2-23.1 %), and unknown taxa (0.3-88 %) were the most abundant taxa from two individuals. | **Sherrill-Mix et al. (2018)** |
|  | Culture | *Photobacterium* *damselae*,*Vibrio* *furnissii*, *Vibrio* *harveyi*, *Moraxella-Pasteurella* sp., and *Alteromonas* sp. detected in the gastrointestinal tract. | **Grimes et al. (1993)** |
| Winter Skate (*Leucoraja ocellata)* | V1-V2 region of 16S rRNA | Unknown taxa (81.8%) and *Photobacterium* sp. (8.2%) were most abundant taxa from one individual. | **Sherrill-Mix et al. (2018)** |
| Yellow stingray (*Urobatis jamaicensis*) | V4 region of 16S rRNA | Microbial community structure was different between cloacal swabs of wild-caught/aquarium-housed and aquarium-born individuals. | **Pinnell et al., (2020)** |
|  |  | *Photobacterium* and *Vibrio* sp. were the most abundant Vibrionaceae taxa observed from wild, wild-caught/aquarium housed, and aquarium-born rays. |  |
|  |  | Vibrionaceae abundance was ~80% vs ~18% in wild vs aquarium-housed rays. |  |

**Supplemental Table 2:  Oral-associated microbes**

| Species | Method | Finding | Source |
| --- | --- | --- | --- |
| Blacktip shark (*Carcharhinus limbatus*) | Culture | *Vibrio alginolyticus* cultured from the oral cavity. | **Grimes et al. (1985)** |
|  | Culture | *Vibrio* *alginolyticus* and *Vibrio* *parahaemolyticus* cultured from teeth. | **Buck (1990)** |
|  | Culture | *Vibrio* *alginolyticus* and *Vibrio* sp. cultured from the teeth. | **Grimes et al. (1993)** |
|  | Culture/API kits | High occurrence (67.9 %) of members of the *Enterobacteriace* family. | **Interaminense et al. (2010)** |
| Bull shark (*Carcharhinus leucas*) | Culture | *Vibrio* *alginolyticus* cultured from teeth. | **Buck (1990)** |
|  | Culture/Siemens Microscan Walk-Away 96 SI | Dominated by gram-negative bacteria, including *Vibrio* *alginolyticus* (14%), other *Vibrio* sp. (14%), and *Pasteurella* sp. (12%), and gram-positive bacteria such as *Staphylococcus* sp. (24%) and *Bacillus* sp. (6%). | **Unger et al. (2014)** |
|  |  | No observed differences between culturable bacterial load and gender or fork length. |  |
| Caribbean reef shark (*Carcharhinus perezi*) | V4 region of 16S rRNA | Teeth microbiomes appear species-specific, likely based on differences in diet and feeding ecologies. | **Storo et al. (2021)** |
| Lemon shark (*Negaprion brevirostris*) | Culture | *Vibrio* *alginolyticus* cultured from the fetal oral cavity. | **Grimes et al. (1985)** |
|  | Culture | *Vibrio* *alginolyticus* and *Vibrio* *parahaemolyticus* cultured from teeth. | **Buck (1990)** |
|  | Culture | *Photobacterium* sp., *Vibrio* *alginolyticus*, *Vibrio* *harveyi*, and *Vibrio* sp. cultured from the oral cavity. | **Grimes et al. (1993)** |
|  | V4 region of 16S rRNA | Contained a high abundance of *Vibrio* (10.8± 26%) and *Corynebacterium* (1.6 ± 5.1%) genera. The genus *Haemophilus* explained 7% of the difference between lemon and Caribbean reef sharks. | **Storo et al. (2021)** |
| Leopard shark (*Triakis semifasciata*) | Culture/API kits | Authors did not distinguish between species within results or multiple body locations were studied confounding interpretation. | **Auerbach et al. (1987)** |
| Nurse shark (*Ginglymostoma cirratum*) | Culture | *Photobacterium* sp. and *Vibrio* *alginolyticus* cultured from oral cavity. | **Grimes et al. (1985)** |
|  | Culture | *Vibrio* *alginolyticus* cultured from teeth. | **Buck (1990)** |
|  | V4 region of 16S rRNA | Teeth microbiomes appear species-specific, likely based on differences in diet and feeding ecologies. | **Storo et al. (2021)** |
|  |  | Contained a high abundance of *Vibrio* (2.8±6.3%) and *Kordia* (3.1±6%) genera as well as *Salmonella enterica* (2.6±6.4%)*.* |  |
| Sandbar shark (*Carcharhinus plumbeus*) | Culture | *Vibrio* *alginolyticus* and *Vibrio* *parahaemolyticus* cultured from teeth. | **Buck (1990)** |
|  | V4 region of 16S rRNA | Teeth microbiomes appear species-specific, likely based on differences in diet and feeding ecologies. | **Storo et al. (2021)** |
|  |  | Contained a high abundance (54±46%) of *Vibrio* genera. |  |
| Atlantic sharpnose shark (*Rhizoprionodon terraenovae*) | Culture | *Photobacterium* sp., *Vibrio* *alginolyticus* and *Vibrio furnissii* cultured from oral cavity. | **Grimes et al. (1985)** |
| Shortfin mako (*Isurus oxyrinchus*) | Culture/API kits | Authors did not distinguish between species within results or multiple body locations were studied confounding interpretation. | **Auerbach et al. (1987)** |
| Spinner shark (*Carcharhinus brevipinna*) | Culture | *Vibrio* *alginolyticus* cultured from teeth. | **Buck (1990)** |
| Tiger shark (*Galeocerdo cuvier*) | Culture | *Proteus* sp. cultured from the oral cavity. | **Grimes et al. (1985)** |
|  | Culture | *Vibrio* *alginolyticus* and *Vibrio* sp. cultured from the teeth. | **Grimes et al. (1993)** |
|  | Culture/Siemens Microscan Walk-Away 96 SI | Dominated by gram-negative bacteria, including *Vibrio* *alginolyticus* (14%), other *Vibrio* sp. (14%), and *Pasteurella* sp. (12%), and gram-positive bacteria such as *Staphylococcus* sp. (24%) and *Bacillus* sp. (6%). | **Unger et al. (2014)** |
|  |  | No observed differences between culturable bacterial load and gender or fork length. |  |
|  | V4 region of 16S rRNA | Teeth microbiomes appear species-specific, likely based on differences in diet and feeding ecologies. | **Storo et al. (2021)** |
|  |  | Contained a high abundance (5.8±12.3%) of *Vibrio* genera. |  |
| White shark (*Carcharodon carcharias*) | Culture | *Citrobacter* sp., *Micrococcus* sp., *Pseudomonas* *putrefaciens*, *Pseudomonas* sp., *Staphylococcus* sp., *Vibrio* *alginolyticus*, *Vibrio* *fluvialis*, *Vibrio* *parahaemolyticus* cultured from teeth. | **Buck et al. (1984)** |
| Clearnose skate (*Raja eglanteria*) | Culture | *Vibrio* *alginolyticus* cultured from teeth. | **Buck (1990)** |
| Southern stingray (*Hypanus americanus*) | Culture | *Vibrio* *alginolyticus* cultured from teeth. | **Buck (1990)** |

**Supplemental Table 3: Skin/Mucus/External-associated microbes**

| Species | Method | Finding | Source |
| --- | --- | --- | --- |
| Blacktip reef shark (*Carcharhinus melanopterus*) | V5-V6 region of 16S rRNA | Dominated by Rhodobacteraceae, Alteromonadaceae, and Halomonadaceae. | **Pogoreutz et al. (2019)** |
|  |  | No microbial differences between visibly healthy skin and skin insulted with injuries. However, there were differences among capture sites suggesting conservation of microbiome under injury and location-specific patterns. |  |
|  | Culture | *Photobacterium* *damselae*, *Staphylococcus* *epidermidis*, *Chryseomonas* *youngae, Streptococcus* alpha, and *Chryseomonas* *luteola* were most abundant. | **Mylniczenko et al. (2007)** |
| Caribbean reef shark (*Carcharhinus perezi*) | V4 region of 16S rRNA | Species was less important than anatomical location in explaining variability in richness, diversity, and composition. All anatomical locations were significantly different from one another with the exception of skin and cloaca. | **Storo et al. (2021)** |
| Japanese wobbegong (*Orectolobus japonicus*) | Culture | *Vibrio* *alginolyticus*, *Photobacterium* *damselae*, *Staphylococcus* *epidermidis*, and *Moraxella* sp. were most abundant. | **Mylniczenko et al. (2007)** |
| Leopard shark (*Triakis semifasciata*) | Culture/API kits | Authors did not distinguish between species within results or multiple body locations were studied confounding interpretation. | **Auerbach et al. (1987)** |
|  | Shotgun Metagenomics | Alphaproteobacteria (37.5%), Deltaproteobacteria (10.8%), Actinobacteria (8.6%), Halobacteria (8.2%), and Gammaproteobacteria (5%) were main components of the microbiome. Patterns of phylosymbiosis are apparent in elasmobranch but not teleost microbiomes. | **Doane et al. (2021)** |
| Lemon shark (*Negaprion brevirostris*) | V4 region of 16S rRNA | Species was less important than anatomical location in explaining variability in richness, diversity, and composition. All anatomical locations were significantly different from one another with the exception of skin and cloaca. | **Storo et al. (2021)** |
| Nurse shark (*Ginglymostoma cirratum*) | V4 region of 16S rRNA | Species was less important than anatomical location in explaining variability in richness, diversity, and composition. All anatomical locations were significantly different from one another with the exception of skin and cloaca. | **Storo et al. (2021)** |
| Sandbar shark (*Carcharhinus plumbeus*) | V4 region of 16S rRNA | Species was less important than anatomical location in explaining variability in richness, diversity, and composition. All anatomical locations were significantly different from one another with the exception of skin and cloaca. | **Storo et al. (2021)** |
|  | Culture | *Photobacterium* *damselae*, *Vibrio* *alginolyticus*, *Pasteurella* *haemolytica*, and *Pseudomonas* *aeruginosa* were most abundant. | **Mylniczenko et al. (2007)** |
| Sandtiger (*Carcharias taurus*) | Culture | Skin dominated by *Micrococcus* sp. (28.5%), *Coryneforms* (28.5%), and *Baccilus* sp. (25.7%). | **Venkataraman and Sreenivasan (1955)** |
| Shortfin mako (*Isurus oxyrinchus*) | Culture/API kits | Authors did not distinguish between species within results or multiple body locations were studied confounding interpretation. | **Auerbach et al. (1987)** |
| Common thresher (*Alopias vulpinus*) | Shotgun Metagenomics | Dominated by *Pseudoalteromonas* (12.7%), *Erythobacter* (5.3%), *Idiomarine* (4.2%), *Limnobacter* (4.1%), and *Marinebacter* (3.8%). | **Doane et al. (2017)** |
|  |  | Cobalt-zinc-cadmium resistance (2.2%), serine-glyoxylate cycle (1.9%), YgfZ system (1.8%), ton/tol transport system (1.3%), and DNA replication (1.2%) were most common gene pathways. |  |
|  |  | Gammaproteobacteria (35.7%) was a major component of the microbiome. Patterns of phylosymbiosis are apparent in elasmobranch but not teleost microbiomes. | **Doane et al. (2021)** |
| Tiger shark  (*Galeocerdo cuvier*) | V4 region of 16S rRNA | Species was less important than anatomical location in explaining variability in richness, diversity, and composition. All anatomical locations were significantly different from one another with the exception of skin and cloaca. | **Storo et al. (2021)** |
| Whale shark (*Rhincodon typus)* | Shotgun Metagenomics | Gammaproteobacteria (60.3%) and Alphaproteobacteria (30.8%) were major compnents of the microbiome. Patterns of phylosymbiosis are apparent in elasmobranch but not teleost microbiomes. | **Doane et al. (2021)** |
| Whitetip reef shark (*Triaenodon obesus*) | Culture | *Photobacterium* *damselae*, *Moraxella* sp., *Pastuerella* *haemolytica*, *Staphylococcus* *epidermidis*, and *Vibrio* *alginolyticus* were most abundant. | **Mylniczenko et al. (2007)** |
| Zebra shark (*Stegostoma fasciatum*) | Culture | *Pseudomonas* *aeruginosa*, *Photobacterium* *damselae*, *Pseudomonas* *fluorescens*, *Vibrio* *alginolyticus*, and *Chryseobacterium* *indologenes* were most abundant. | **Mylniczenko et al. (2007)** |
| Lesser devil ray (*Mobula hypostoma*) | Culture | 18.1% of bacterial isolates showed antimicrobial activity. | **Ritchie et al. (2017)** |
| Atlantic stingray (*Dasyatis sabina*) | Culture | Difference between marine and freshwater Atlantic stingrays; suggesting a role of environment in shaping skin microbiomes. | **Ritchie et al. (2017)** |
|  |  | 18.6 % and 9.4% of bacterial isolates from marine and freshwater individuals showed antimicrobial resistance, respectively. |  |
| Groovebelly ray (*Dasyatis hypostigma*) | V4 region of 16S rRNA | Bacterial community changed with age of *D. hypostigma*. | **Goncalves e Silva et al. (2020)** |
|  |  | *Corynebacterium* and *Staphylococcus* were main contributors to dissimilarity between newborns and adults. |  |
|  |  | A predominance of *Oceanimonas* (25.8%) was observed in adults. |  |
| Cownose ray (*Rhinoptera bonasus*) | V1-V9 region of 16S rRNA | Dominated by *Burkholderiales* (55%), *Flavobacteriales* (19%), and *Pseudomonadales* (12%). | **Kearns et al. (2017)** |
|  |  | No changes were observed after contact with humans. |  |
|  | Culture | 20.8% of bacterial isolates showed antimicrobial activity. | **Ritchie et al. (2017)** |
| Butterfly ray (*Gymnura altavela*) | V4 region of 16S rRNA | Dominant phyla from skin and stinger of *G. atavela* in natural settings were Proteobacteria, Bacteroidetes, and Actinobacteria. Dominant phyla from skin in an aquarium setting was dominated by Proteobacteria (~86%).  Stinger samples were dominated by Bacteroidetes (~53%) and Proteobacteria (~44%) in aquaria. | **Goncalves e Silva et al. (2020)** |
|  |  | High number of taxonomic groups were shared between *G. altavela*  and sediment highlighting the influence of sediment on ray microbiomes. |  |
| Ocellate spot skate (*Okamejei kenojei*) | Biolog Microstation System after rinse bag method | *Photobacterium* sp. and *Vibrio* sp. were most abundant. | **Cho et al. (2004)** |
| Round ray (*Urolophus helleri*) | Shotgun Metagenomics | Alphaproteobacteria (69.5%) and Gammaproteobacteria (153%) were major components of microbiome. Patterns of phylosymbiosis are apparent in elasmobranch but not teleost microbiomes. | **Doane et al. (2021)** |
| Clearnose skate (*Raja eglanteria*) | Culture | 2.4% of bacterial isolates showed antimicrobial activity. | **Ritchie et al. (2017)** |
| Skate (*Raja* sp.) | Culture | Mucus dominated by *Pseudomonas* sp. (63%). | **Liston (1957)** |
| Yellow stingray (*Urobatis jamaicensis*) | V4 region of 16S rRNA | Microbial community structure was different between gills and skin of wild and aquarium-housed individuals. | **Pinnell et al. (2020)** |
|  |  | *Vibrio* was the most dominant taxa in gill and skin associated communities. |  |
|  |  | A decrease in relative abundance of Bacteroidetes and increase in the relative abundance of Proteobacteria was observed between wild and aquarium-housed rays. Differences were primarily driven by a lower abundance of Saprospiraceae. |  |

**Supplemental Table 4: Internal tissue-associated microbes**

| Species | Method | Findings | Source |
| --- | --- | --- | --- |
| Blacktip shark (*Carcharhinus limbatus*) | Culture | *Vibrio parahaemolyticus* and *Vibrio* sp. cultured from blood. | **Grimes et al. (1985)** |
|  | Culture | *Photobacterium* *damselae* and *Vibrio* *alginolyticus* detected in blood. *Photobacterium* *damselae*, *Vibrio* *alginolyticus*, *Alteromonas* sp., *Vibrio* *harveyi*, and *Vibrio* sp. detected in internal organs. | **Grimes et al. (1993)** |
|  | Culture | Three bacterial species isolated. *Photobacterium* *damselae*, *Vibrio* *alginolyticus*, and *Vibrio* *parahaemolyticus* cultured from blood. | **Mylniczenko et al. (2007)** |
| Blacktip reef shark (*Carcharhinus melanopterus*) | Culture | Sixteen bacterial species isolated. *Photobacterium* *damselae*, *Citrobacter* *youngae*,*Staphylococcus* *epidermidis*, and *Citrobacter* *freundii* were most abundant species cultured from blood. | **Mylniczenko et al. (2007)** |
| Blacknose shark (*Carcharhinus acronotus*) | Culture | One bacterial species isolated. *Staphylococcus* *epidermidis* cultured from blood. | **Mylniczenko et al. (2007)** |
| Japanese wobbegong (*Orectolobus japonicus*) | Culture | One bacterial species isolated. *Photobacterium* *damselae* cultured from blood. | **Mylniczenko et al. (2007)** |
| Leopard shark (*Triakis semifasciata*) | Culture | One bacterial species isolated. *Vibrio* *alginolyticus* cultured from blood. | **Mylniczenko et al. (2007)** |
| Lemon shark (*Negaprion brevirostris*) | Culture | *Vibrio alginolyticus* and *Vibrio* sp. cultured from blood. | **Grimes et al. (1985)** |
|  | Culture | *Vibrio* *alginolyticus* and *Vibrio* sp. detected in blood. *Aeromonas* *salmonicida*, *Moraxella-Pasteurella* sp., *Photobacterium* *damselae*, and *Vibrio* *alginolyticus* detected in internal organs. | **Grimes et al. (1993)** |
| Narrowsnout sawfish (*Pristis zijsron*) | Culture | One bacterial species isolated. *Pasteurella* *pneumotropica* cultured from blood. | **Mylniczenko et al. (2007)** |
| Nurse shark (*Ginglymostoma cirratum*) | Culture | *Vibrio* *harveyi* cultured from liver, spleen, and pancreas. | **Grimes et al. (1985)** |
|  | Culture | *Photobacterium* *damselae* and *Photobacterium* sp. detected in blood. | **Grimes et al. (1993)** |
| Sandbar shark (*Carcharhinus plumbeus*) | Culture | Two bacterial species isolated. *Staphylococcus* *epidermidis* and *Vibrio* *vulnificus* cultured from blood. | **Mylniczenko et al. (2007)** |
| Atlantic sharpnose shark (*Rhizoprionodon terraenovae*) | Culture | *Vibrio* sp. cultured from the liver. | **Grimes et al. (1985)** |
| Smooth dogfish (*Mustelus canis*) | Culture | *Vibrio* sp., *Alteromonas* sp., *Shewanella* sp., *Photobacterium* sp., and *Pseudomonas* sp. were cultured from the kidney. | **Borucinska and Frasca (2002)** |
| Spiny dogfish (*Squalus acanthias*) | Culture | *Vibrio* sp., *Alteromonas* sp., *Shewanella* sp., *Photobacterium* sp., and *Pseudomonas* sp. were cultured from the kidney. | **Borucinska and Frasca (2002)** |
| Swell shark (*Cephaloscyllium ventriosum*) | Culture | Two bacterial species isolated. *Staphylococcus* *epidermidis* and *Stenotrophomonas* *maltophilia* cultured from blood. | **Mylniczenko et al. (2007)** |
| Tiger shark *(Galeocerdo cuvier*) | Culture | *Vibrio* *alginolyticus* detected in the liver. | **Grimes et al. (1993)** |
| Whitetip reef shark (*Triaenodon obesus*) | Culture | Seven bacterial species cultured. *Photobacterium* *damselae*, *Pasteurella* *pneumotropica*, *Staphylococcus* *epidermidis* were cultured from blood. | **Mylniczenko et al. (2007)** |
| Whitespotted bamboo shark (*Chiloscyllium plagiosum*) | Culture | Two bacterial species isolated. *Vibrio* *vulnificus* and *Vibrio* *alginolyticus* cultured from blood. | **Mylniczenko et al. (2007)** |
| Lesser electric ray (*Narcine bancroftii*) | 16S rRNA gene sequencing V1-V8 region | Blood was positive for bacteria in 90% of samples. The majority of the isolates belonged to the phylum Proteobacteria (91.5%). | **Tao et al. (2014)** |
|  |  | *Vibrio* sp. comprised 53% of all isolates and were recovered from all samples. *V. harveyi* (n = 14) and *V. campbellii* (n = 11) were most common, followed by a group of unidentified *Vibrio sp.* (n = 10) related to *V. nigripulchritudo.* |  |
| Mangrove whiptail ray (*Urogymnus granulatus*) | Culture | Five bacterial species isolated. *Vibrio* *alginolyticus*, *Morganella* *morganii*, *Photobacterium* *damselae*, *Vibrio* *vulgaris,* and *Proteus* *vulgaris* were cultured from blood. | **Mylniczenko et al. (2007)** |
| Freshwater stingray (*Potamotrygon* spp.) | Culture | Four bacterial species isolated. *Vibrio* *fluvialis*, *Plesiomonas* *shigelloides*, *Staphylococcus* *epidermidis*, and *Pseudomonas* *fluorescens*/*putida* cultured from blood. | **Mylniczenko et al. (2007)** |

Literature Cited

Auerbach, P.S., Yajko, D.M., Nassos, P.S., Kizer, K.W., McCosker, J.E., Geehr, E.C., and Hadley, W.K. (1987) Bacteriology of the marine environment: implications for clinical therapy. *Annals of emergency medicine* **16**(6), 643-649.

Borucinska, J., and Frasca, S. (2002) Naturally occurring lesions and micro‐organisms in two species of free‐living sharks: the spiny dogfish, *Squalus acanthias* L., and the smooth dogfish, *Mustelus canis* (Mitchill), from the north‐western Atlantic. *Journal of Fish Diseases* **25**(5), 287-298.

Buck, J.D. (1990) Potentially pathogenic marine Vibrio species in seawater and marine animals in the Sarasota, Florida, area. *Journal of coastal research*, 943-948.

Buck, J.D., Spotte, S., and Gadbaw Jr, J. (1984) Bacteriology of the teeth from a great white shark: potential medical implications for shark bite victims. *Journal of Clinical Microbiology* **20**(5), 849-851.

Cho, S.-h., Jahncke, M.L., and Eun, J.-b. (2004) Nutritional composition and microflora of the fresh and fermented skate (*Raja Kenojei*) skins. *International journal of food sciences and nutrition* **55**(1), 45-51.

Doane, M.P., Haggerty, J.M., Kacev, D., Papudeshi, B., and Dinsdale, E.A. (2017) The skin microbiome of the common thresher shark (*Alopias vulpinus*) has low taxonomic and gene function β‐diversity. *Environmental microbiology reports* **9**(4), 357-373.

Doane, M.P., Morris, M.M., Papudeshi, B., Allen, L., Pande, D., Haggerty, J.M., Johri, S., Turnlund, A.C., Peterson, M., and Kacev, D. (2020) The skin microbiome of elasmobranchs follows phylosymbiosis, but in teleost fishes, the microbiomes converge. *Microbiome* **8**.

Givens, C.E., Ransom, B., Bano, N., and Hollibaugh, J.T. (2015) Comparison of the gut microbiomes of 12 bony fish and 3 shark species. *Marine Ecology Progress Series* **518**, 209-223.

Goncalves e Silva, F., Dos Santos, H.F., de Assis Leite, D.C., Lutfi, D.S., Vianna, M., and Rosado, A.S. (2020) Skin and stinger bacterial communities in two critically endangered rays from the South Atlantic in natural and aquarium settings. *MicrobiologyOpen* **9**(12), e1141.

Grimes, D., Brayton, P., Colwell, R., and Gruber, S. (1985) Vibrios as autochthonous flora of neritic sharks. *Systematic and Applied Microbiology* **6**(2), 221-226.

Grimes, D.J., Jacobs, D., Swartz, D., Brayton, P., and Colwell, R.R. (1993) Numerical taxonomy of gram-negative, oxidase-positive rods from carcharhinid sharks. *International Journal of Systematic and Evolutionary Microbiology* **43**(1), 88-98.

Interaminense, J., Nascimento, D., Ventura, R., Batista, J., Souza, M., Hazin, F., Pontes-Filho, N., and Lima-Filho, J. (2010) Recovery and screening for antibiotic susceptibility of potential bacterial pathogens from the oral cavity of shark species involved in attacks on humans in Recife, Brazil. *Journal of medical microbiology* **59**(8), 941-947.

Johny, T.K., Saidumohamed, B.E., Sasidharan, R.S., and Bhat, S.G. (2018) Metabarcoding data of bacterial diversity of the deep sea shark, Centroscyllium fabricii. *Data in brief* **21**, 1029-1032.

Juste-Poinapen, N.M., Yang, L., Ferreira, M., Poinapen, J., and Rico, C. (2019) Community profiling of the intestinal microbial community of juvenile Hammerhead Sharks (*Sphyrna lewini*) from the Rewa Delta, Fiji. *Scientific reports* **9**(1), 1-11.

Kearns, P.J., Bowen, J.L., and Tlusty, M.F. (2017) The skin microbiome of cow‐nose rays (*Rhinoptera bonasus*) in an aquarium touch‐tank exhibit. *Zoo biology* **36**(3), 226-230.

Leigh, S.C., Papastamatiou, Y.P., and German, D.P. (2021) Gut microbial diversity and digestive function of an omnivorous shark. *Marine Biology* **168**(5), 1-16.

Liston, J. (1957) The occurrence and distribution of bacterial types on flatfish. *Microbiology* **16**(1), 205-216.

Mylniczenko, N.D., Harris, B., Wilborn, R.E., and Young, F.A. (2007) Blood culture results from healthy captive and free-ranging elasmobranchs. *Journal of Aquatic Animal Health* **19**(3), 159-167.

Pinnell, L.J., Oliaro, F.J., and Van Bonn, W. (2020) Host-associated microbiota of yellow stingrays (*Urobatis jamaicensis*) is shaped by their environment and life history. *Marine and Freshwater Research* **72**(5), 658-667.

Pogoreutz, C., Gore, M.A., Perna, G., Millar, C., Nestler, R., Ormond, R.F., Clarke, C.R., and Voolstra, C.R. (2019) Similar bacterial communities on healthy and injured skin of black tip reef sharks. *Animal Microbiome* **1**(1), 1-16.

Ritchie, K.B., Schwarz, M., Mueller, J., Lapacek, V.A., Merselis, D., Walsh, C.J., and Luer, C.A. (2017) Survey of antibiotic-producing bacteria associated with the epidermal mucus layers of rays and skates. *Frontiers in microbiology* **8**, 1050.

Sherrill-Mix, S., McCormick, K., Lauder, A., Bailey, A., Zimmerman, L., Li, Y., Django, J.-B.N., Bertolani, P., Colin, C., and Hart, J.A. (2018) Allometry and ecology of the bilaterian gut microbiome. *Mbio* **9**(2), e00319-18.

Storo, R., Easson, C., Shivji, M., and Lopez, J.V. (2021) Microbiome Analyses Demonstrate Specific Communities Within Five Shark Species. *Frontiers in microbiology* **12**, 139.

Tao, Z., Bullard, S.A., and Arias, C.R. (2014) Diversity of bacteria cultured from the blood of lesser electric rays caught in the northern Gulf of Mexico. *Journal of aquatic animal health* **26**(4), 225-232.

Unger, N.R., Ritter, E., Borrego, R., Goodman, J., and Osiyemi, O.O. (2014) Antibiotic susceptibilities of bacteria isolated within the oral flora of Florida blacktip sharks: guidance for empiric antibiotic therapy. *PLoS One* **9**(8), e104577.

Venkataraman, R., and Sreenivasan, A. (1955) Bacterial flora of fresh shark. *Current Science* **24**(11), 380-381.
